# Lung Cancer Mortality and Serum Levels of Carotenoids, Retinol, Tocopherols, and Folic Acid in Men and Women: a Case-Control Study Nested in the JACC Study

**DOI:** 10.2188/jea.15.S140

**Published:** 2005-08-18

**Authors:** Yoshinori Ito, Kenji Wakai, Koji Suzuki, Kotaro Ozasa, Yoshiyuki Watanabe, Nao Seki, Masahiko Ando, Yoshikazu Nishino, Takaaki Kondo, Yoshiyuki Ohno, Akiko Tamakoshi

**Affiliations:** 1 Department of Public Health, Fujita Health University School of Health Sciences.; 2 Division of Epidemiology and Prevention, Aichi Cancer Center Research Institute.; 3 Department of Epidemiology for Community Health and Medicine, Kyoto Prefectural University of Medicine, Graduate School of Medical Science Division of Public Health.; 4 Department of Infectious Disease Control and International Medicine, Niigata University Graduate School of Medical and Dental Science.; 5 Kyoto University Center for Student Health.; 6 Division of Epidemiology, Department of Public Health and Forensic Medicine, Tohoku University Graduate School of Medicine.; 7 Department of Medical Technology, Nagoya University School of Health Sciences.; 8 Department of Preventive Medicine/Biostatistics and Medical Decision Making, Nagoya University Graduate School of Medicine.

**Keywords:** Lung Neoplasms, nested case-control study, beta-carotene, beta-cryptoxanthin, lycopene

## Abstract

BACKGROUND: Lung cancer mortality is inversely associated with high serum carotenoid levels and high intake of vegetables and fruits rich in carotenoids. The Japan Collaborative Cohort (JACC) Study was conducted to investigate whether serum levels of carotenoids, retinol, tocopherols, and folic acid were associated with risk for lung cancer death with follow-up through 1997. To examine the association by sex, we extended the follow-up and analyzed additional serum samples.

METHODS: In the JACC Study, 39,242 subjects provided serum samples at baseline between 1988 and 1990. We identified 211 cases (163 men and 48 women) of death from lung cancer during about 10-year follow-up ending in 1999. Of the subjects who survived to the end of that follow-up, 487 controls (375 men and 112 women) were selected, and were matched to each case of lung cancer death for sex, age and participating institution. We measured serum levels of antioxidants in cases of lung cancer death and controls. Odds ratio (OR) for lung cancer death was estimated using conditional logistic models by sex.

RESULTS: For men, the risk of lung cancer death was significantly lower for the highest quartile of serum *α*- and *β*-carotenes, lycopene, and *β*-cryptoxanthin than for the lowest quartile: the OR adjusted for smoking and other covariates were 0.41, 0.28, 0.46, and 0.39, respectively. For women, serum levels of *α*-carotene and zeaxanthin/lutein were inversely associated with risk of lung cancer, but the association was not significant. No association between lung cancer and serum levels of *β*-carotene, *β*-cryptoxanthin, and retinol was appeared among women. There was a suggestion that association between lung cancer and high serum levels of these components might differ between men and women.

CONCLUSIONS: Higher serum levels of carotenoids appear to play a role in preventing death from lung cancer among Japanese men. Relationships between lung cancer and serum levels of some carotenoids appear to differ between sexes. However, further study with a large number of women cases needs to clarify the discrepancy between sexes.

Many epidemiological studies have shown that dietary intake of vegetables and fruits rich in carotenoids such as *β*-carotene (BC) can decrease human lung cancer risk.^1^^-^^6^ Recent studies have found that serum carotenoids such as *β*-cryptoxanthin (CR) and lutein have clear inverse associations with lung cancer risk.^7^ We previously found that high serum levels of *α*-carotene (AC) and BC can reduce the risk of lung cancer death, in a case-control study nested in a large-scale Japanese cohort.^8^ Serum levels of AC and BC are affected by daily lifestyle elements such as diet, smoking and alcohol consumption,^9^^,^^10^ and this appears to be partly responsible for sex differences in lung cancer risk. Recent intervention studies of BC administration have shown no inverse association between BC administration and lung cancer incidence among non-Asians.^4^^,^^11^^-^^13^

In the present study, to examine the association by sex, we extended the follow-up until 1999 and analyzed additional serum samples. We investigated the association between death from lung cancer and serum levels of carotenoids and other antioxidants in Japanese.

## METHODS

### Subjects and Blood Samples

The study subjects were recruited in the Japan Collaborative Cohort Study (JACC Study) for Evaluation of Cancer Risk sponsored by the Ministry of Education, Science, Sports, and Culture of Japan (Monbusho). The details of the JACC study are described elsewhere.^14^^,^^15^ A questionnaire about health and lifestyle was distributed to the participants. The questions addressed personal and family medical histories, smoking, alcohol consumption, dietary intake of major foods, and anthrometric factors.^14^^,^^15^ In addition to completing the questionnaire survey, participants in the present study gave peripheral blood samples at health screening check-ups sponsored by municipalities between 1988 and 1990. A total of 39,242 subjects aged 40 to 79 years (35.3% of the 110,792 respondents to the questionnaire survey) provided blood samples. Sera were separated from the samples at laboratories in or near the surveyed municipalities as soon as possible after the blood was drawn.^16^ Serum of each participant was divided into 3 to 5 tubes (100 to 500 *μ*L per tube), and serum samples were stored in deep freezers at -80°C until 2001 and 2002. We found the following decreases in mean percentages of serum components using pooled sera after 9 years of storage at -80°C: AC and BC, less than 10% decrease; lycopene (LY), CR, zeaxanthin&lutein (ZL), retinol (RE), and *α*-tocopherol (AT), less than 20%.

Informed consent for participation was obtained individually from subjects, with the exception of those in some study areas in which informed consent was provided as the group level after the aim of the study and confidentiality of the data had been explained to community leaders. This study was approved by the Ethical Board of the Nagoya University School of Medicine.

### Selection of Cases and Controls

Lung cancer deaths were identified using municipal registration records and death certificates.^14^^,^^15^ We followed subjects until late 1999, and eligible case subjects were defined as those who died from lung cancer (International Statistical Classification of Diseases and Related Health Problems 10th Revision: C34). During about 10-year follow-up, 240 deaths from lung cancer were identified among the subjects who provided baseline serum samples. Of these, we excluded 8 subjects with a previous history of cancer, and excluded 21 who lacked suitable samples. For each case of lung cancer death (hereafter referred to as “cases”), 2 or 3 controls were selected from the survivors, matching for sex, age (as near as possible), and participating institution. Of the initial 569 matched controls, we excluded 6 with a previous history of cancer, 30 who lacked sufficient samples, and 46 whose matched cases were excluded. Thus, 211 cases and 487 controls were included in the analysis.

### Determination of Serum Carotenoids, Retinol, Tocopherols, and Folic Acid

All samples were analyzed by trained staff blinded to case-control status. Serum total cholesterol (TC) was determined using an autoanalyzer. Serum concentrations of carotenoids, RE, and tocopherols were measured by high-performance liquid chromatography, as described elsewhere.^17^ All samples were stored in deep freezers at -80°C for about 11 years, and serum levels of carotenoids, RE and tocopherols were measured using the same equipment for all specimen; the ranges of repeatability and day-to-day variation were 4.6% to 6.9% and 6.3% to 20.0%, respectively, for the assays of carotenoids, RE, and tocopherols.^17^ We could not separately measure serum levels of zeaxanthin and lutein or *β*- and *γ*-tocopherols, and therefore report the combined levels as ZL or *β*- and *γ*-tocopherols (BT), respectively. We calculated total carotenes (TCR) as the sum of AC, BC, and LY, calculated total xanthophylls (TXP) as the sum of CR, ZL, and canthaxanthin (CX), and calculated total provitamin A (PVA) as the sum of AC, BC, and CR. Total carotenoids (TCA) were calculated as TCR plus TXP. Serum folic acid levels were determined using a cloned enzyme donor immunoassay.^18^

### Statistical Analysis

Body mass index (BMI) at baseline was calculated from reported height and weight: BMI = (weight in kg)/(height in m)^2^. Mean differences between lung cancer cases and matched controls were examined by t test after converting serum levels of carotenoids, RE, tocopherols, folic acid and TC to logarithmic values. Analysis of covariance (ANCOVA) was also performed after controlling for age, participating institutions, smoking, alcohol consumption, BMI and serum TC level.^19^ Two conditional logistic regression models were used to calculate odds ratio (OR) for lung cancer death.^19^ Variables adjusted in the models were as follows: model 1 (OR1), age, participating institution, and smoking; model 2 (OR2), age, participating institution, smoking, alcohol consumption, BMI, and serum TC level.

In both sexes, cases and controls were categorized into 4 groups according to quartile levels of carotenoids, RE, tocopherols, folic acid, and TC: Q1 (lowest) to Q4 (highest). However, the control subjects were not precisely divided into 4 equal groups, because some controls had identical serum values. The OR was calculated for Q2, Q3 and Q4 versus Q1. To test for linear trends across the quartiles, we coded each quartile as 0, 1, 2, or 3 and then incorporated it into the logistic model as a single variable. Because smoking is a very important risk factor for lung cancer, it was adjusted using the following detailed strata for men: subjects who had never smoked; former smokers who had not smoked for 0 to 4 years, 5 to 9, 10 to 14, 15 to 19, or 20 y or longer; and current smokers with pack-years of 0 to 19, 20 to 39, 40 to 59, 60 to 79, 80 to 99, or 100 or more. For women, subjects were categorized by smoking status (i.e., never, former, or current smokers). All p values were two-sided, and all analyses were performed using the Statistical Analysis System.^19^

## RESULTS

### Subject Characteristics

Table 1 shows the distribution of study subjects by sex, age, smoking, alcohol consumption, BMI and some medical history at baseline. About half of the subjects were in their 60s years of age, and about 30% were in their 70s. For both sexes, current smokers comprised a much greater proportion of cases than controls, whereas current drinkers comprised a slightly smaller proportion of cases than controls. A greater proportion of women than men had high BMI (25.0+ kg/m^2^), and a greater proportion of controls than cancer cases had high BMI. There was no apparent difference in major disease history between cases and controls.

**Table 1.** Baseline characteristics of 211 lung cancer cases and 487 controls.

Characteristics		Male				Female				Total			
		
		Cases (%)		Controls (%)		Cases (%)		Controls (%)		Cases (%)		Controls (%)	
Total		163	(100.0)	375	(100.0)	48	(100.0)	112	(100.0)	211	(100.0)	487	(100.0)

Age (year)	40-49	3	(1.8)	8	(2.1)	3	(6.3)	6	(5.4)	6	(2.8)	14	(2.9)
	50-59	20	(12.3)	51	(13.6)	12	(25.0)	29	(25.9)	32	(15.2)	80	(16.4)
	60-69	89	(54.6)	219	(58.4)	22	(45.8)	49	(43.8)	111	(52.6)	268	(55.0)
	70-79	51	(31.3)	97	(25.9)	11	(22.9)	28	(25.0)	62	(29.4)	125	(25.7)

Smoking status	Current smoker	115	(70.6)	176	(46.9)	5	(10.4)	5	(4.5)	120	(56.9)	181	(37.2)
	Former smoker	36	(22.1)	104	(27.7)	0	(0.0)	2	(1.8)	36	(17.1)	106	(21.8)
	Has never smoked	5	(3.1)	83	(22.1)	37	(77.1)	93	(83.0)	42	(19.9)	176	(36.1)
	Unknown	7	(4.3)	12	(3.2)	6	(12.5)	12	(10.7)	13	(6.2)	24	(4.9)

Alcohol consumption	Current drinker	104	(63.8)	254	(67.7)	7	(14.6)	23	(20.5)	111	(52.6)	277	(56.9)
	Former drinker	18	(H.0)	17	(4.5)	0	(0.0)	2	(1.8)	18	(8.5)	19	(3.9)
	Has never consumed alcohol	37	(22.7)	93	(24.8)	38	(79.2)	80	(71.4)	75	(35.5)	173	(35.5)
	Unknown	4	(2.5)	11	(2.9)	3	(6.3)	7	(6.3)	7	(3.3)	18	(3.7)

Body mass index (kg/m^2^)	<20.0	35	(21.5)	61	(16.3)	8	(16.7)	15	(13.4)	43	(20.4)	76	(15.6)
	20.0-24.9	108	(66.3)	238	(63.5)	29	(60.4)	67	(59.8)	137	(64.9)	305	(62.6)
	25.0-	16	(9.8)	62	(16.5)	8	(16.7)	26	(23.2)	24	(11.4)	88	(18.1)
	Unknown	4	(2.5)	14	(3.7)	3	(6.3)	4	(3.6)	7	(3.3)	18	(3.7)

Medical history (yes)	Myocardial infarction	4	(2.5)	11	(2.9)	1	(2.1)	6	(5.4)	5	(2.4)	17	(3.5)
	Apoplexy	0	(0.0)	6	(1.6)	1	(2.1)	1	(0.9)	1	(0.5)	7	(1.4)
	Liver diseases	14	(8.6)	30	(8.0)	3	(6.3)	10	(8.9)	17	(8.1)	40	(8.2)
	Kidney diseases	4	(2.5)	16	(4.3)	0	(0.0)	5	(4.5)	4	(1.9)	21	(4.3)
	Diabetes	10	(6.1)	23	(6.1)	1	(2.1)	3	(2.7)	11	(5.2)	26	(5.3)

### Comparison of Serum Levels of Carotenoids, Retinol, Tocopherols and Folic Acid between Lung Ccancer Cases and Controls

Serum levels of carotenoids and other components at baseline were compared between lung cancer cases and matched controls (Table 2). For men, serum levels of AC, BC, LY, TCR, CR and RE were significantly lower for lung cancer cases than for matched controls. For women, there were no significant differences in serum levels of carotenoids, RE or tocopherols (except for TC) between cancer cases and controls, although serum levels of BC, LY, TCR, RE, TXP, and TCA tended to be lower for cancer cases. After adjusting for age, participating institution, smoking, alcohol consumption, BMI and serum TC level, we found that for men, serum levels of AC and LY were significantly lower in cancer cases than in controls. Serum levels of BC, TCR, CR, PVA, RE, and TC tended to be lower in cancer cases, especially among men, but the differences were not significant. Among controls, all serum levels of these components other than RE and CX were higher in women than in men.

**Table 2.** Geometric means and 5-95 percentiles (*μ*mol/L) of serum levels of carotenoids, retinol, tocopherols, folic acid, and total cholesterol in lung cancer cases and controls.

	Male						Female					
	
	Geometric mean (5-95 percentile)				P for difference		Geometric mean (5-95 percentile)				P for difference	
			
	Cases (N = 163)		Controls (N = 375)		Univariate	Multivariate*	Cases (N = 48)		Controls (N = 112)		Univariate	Multivariate*
*α*-Carotene [AC] (*μ*mol/L)	0.041	(0.009 - 0.125)	0.050	(0.012 - 0.157)	0.011	0.043	0.083	(0.023 - 0.272)	0.081	(0.010 - 0.231)	0.85	0.77
*β*-Carotene [BC] (*μ*mol/L)	0.21	(0.04 - 0.77)	0.26	(0.03 - 1.18)	0.042	0.082	0.58	(0.10 - 1.71)	0.64	(0.09 - 2.12)	0.59	0.30
Lycopene [LY] (*μ*mol/L)	0.06	(0.01 - 0.21)	0.07	(0.01 - 0.36)	0.025	0.032	0.10	(0.03 - 0.30)	0.12	(0.03 - 0.51)	0.20	0.33
Total carotenes [TCR] (*μ*mol/L)	0.42	(0.10 - 1.41)	0.50	(0.09 - 1.94)	0.048	0.074	0.94	(0.26 - 2.66)	1.04	(0.18 - 3.13)	0.50	0.34

*β*-Cryptoxanthin [CR] (*μ*mol/L)	0.12	(0.02 - 0.79)	0.15	(0.02 - 0.82)	0.042	0.19	0.30	(0.08 - 1.24)	0.28	(0.04 - 1.17)	0.70	0.87
Zeaxanthin&lutein [ZL] (*μ*mol/L)	0.81	(0.33 - 1.79)	0.85	(0.33 - 2.19)	0.31	0.65	0.91	(0.41 - 2.45)	1.01	(0.37 - 2.36)	0.31	0.060
Canthaxanthin [CX] (*μ*mol/L)	0.029	(0.010 - 0.071)	0.030	(0.010 - 0.078)	0.49	0.67	0.037	(0.016 - 0.094)	0.038	(0.016 - 0.075)	0.70	1.00
Total xanthophylls [TXP] (*μ*mol/L)	1.02	(0.38 - 2.28)	1.09	(0.40 - 2.65)	0.22	0.66	1.33	(0.56 - 2.93)	1.43	(0.51 - 3.24)	0.43	0.15

Total carotenoids [TCA] (*μ*mol/L)	1.50	(0.54 - 3.62)	1.65	(0.49 - 4.22)	0.093	0.27	2.33	(0.74 - 5.45)	2.56	(0.82 - 6.45)	0.38	0.14
Provitamin A [PVA] (*μ*mol/L)	0.41	(0.09 - 1.39)	0.49	(0.07 - 1.91)	0.051	0.12	1.02	(0.19 - 2.53)	1.07	(0.15 - 2.93)	0.73	0.33

Retinol [RE] (*μ*mol/L)	2.43	(1.40 - 3.73)	2.61	(1.56 - 4.15)	0.021	0.19	2.28	(1.32 - 3.60)	2.31	(1.39 - 3.46)	0.85	0.44
*α*-Tocopherol [AT] (*μ*mol/L)	21.48	(13.25 - 39.35)	21.46	(11.09 - 38.56)	0.98	0.57	24.02	(15.91 - 37.47)	24.52	(12.05 - 40.04)	0.77	0.72
*β*- & *γ*-Tocopherols [BT] (*μ*mol/L)	2.69	(0.99 - 5.51)	2.89	(1.21 - 5.69)	0.15	0.18	3.08	(0.60 - 7.83)	3.57	(1.57 - 7.68)	0.12	0.37

Folic acid (ng/mL)^†^	5.21	(2.80 - 12.70)	5.46	(2.50 - 12.90)	0.47	0.81	6.21	(3.30 - 14.60)	6.63	(2.90 - 13.90)	0.53	0.17
Total cholesterol [TC] (mmol/L)^‡^	4.69	(3.16 - 6.47)	4.79	(3.39 - 6.60)	0.23	0.13	4.95	(3.47 - 6.72)	5.31	(3.98 - 7.01)	0.033	0.096

* : P value for difference of the geometric mean between cases and controls adjusted for age, participating institution, smoking, alcohol drinking, body mass index, and serum total cholesterol levels by analysis of covariance.

† : The numbers of cases and controls were 81 and 177 for men and 29 and 63 for women.

‡ : The numbers of cases and controls were 159 and 370 for men and 48 and 108 for women.

### Odds Ratio for Lung Cancer Death

The OR of serum carotenoids and other components for lung cancer mortality is shown in Table 3. For men, risk adjusted for participating institution, age and smoking (model 1: OR1) was significantly lower for the highest serum levels of AC, BC, LY, TCR, CR and RE than for the lowest levels. Also for men, with the exception of RE, TC and folic acid, the OR also tended to be lower for moderate serum levels (Q2 and/or Q3) than for the lowest levels. The OR tended to be lower (though not significantly so) for the highest serum levels of CX, TCA,BT, and AT. For women, the OR tended to be lower for the highest serum levels of carotenoids and other components, with the exception of BC, CR, and RE, but the differences were not significant.

**Table 3.** Odds ratio (OR) and 95% confidence intervals (CI) for lung cancer death by sex and serum levels of carotenoids, retinol, tocophenols, folic acid, and total cholesterol.

	Male									Female								
	
	Category	Cases/ controls		OR1*	95% CI	P for trend	OR2^†^	95% CI	P for trend	Category	Cases/ controls		OR1*	95% CI	P for trend	OR2^†^	95% CI	P for trend
*α*-Carotene [AC] (*μ*mol/L)	<0.032	48	86	1.00	Reference		1.00	Reference		<0.058	12	27	1.00	Reference		1.00	Reference	
	0.032-0.053	52	101	0.89	0.52 - 1.53		0.88	0.49 - 1.58		0.058-0.090	13	29	0.80	0.26 - 2.44		0.54	0.15 -1.89	
	0.054-0.089	37	93	0.66	0.35 - 1.23		0.60	0.31 - 1.18		0.091-0.149	14	27	0.88	0.25 - 3.11		0.77	0.18 - 3.30	
	0.090-	26	95	0.41	0.20 - 0.85	0.01	0.40	0.18 - 0.86	0.02	0.150-	9	29	0.52	0.13 - 2.06	0.38	0.39	0.07 - 2.1	0.41

*β*-Carotene [BC] (*μ*mol/L)	<0.14	47	93	1.00	Reference		1.00	Reference		<0.40	12	28	1.00	Reference		1.00	Reference	
	0.14-0.29	55	94	0.78	0.43 - 1.44		0.78	0.41 - 1.51		0.40-0.74	18	28	1.50	0.50 - 4.49		1.42	0.41 - 4.91	
	0.30-0.57	42	93	0.73	0.39 - 1.37		0.71	0.36 - 1.39		0.75-1.20	5	28	0.43	0.11 - 1.72		0.20	0.04 - 1.15	
	0.58-	19	95	0.28	0.13 - 0.64	0.01	0.23	0.09 - 0.55	0.00	1.21-	13	28	0.97	0.27 - 3.43	0.49	0.82	0.19 - 3.58	0.48

Lycopene [LY] (*μ*mol/L)	<0.04	46	91	1.00	Reference		1.00	Reference		<0.07	12	28	1.00	Reference		1.00	Reference	
	0.04-0.06	48	87	1.03	0.58 - 1.81		1.07	0.58 - 1.95		0.07-0.11	15	28	1.24	0.47 - 3.24		1.17	0.35 - 3.96	
	0.07-0.14	45	103	0.71	0.38 - 1.33		0.59	0.30 - 1.17		0.12-0.19	12	28	1.09	0.39 - 3.04		0.92	0.25 - 3.42	
	0.15-	24	94	0.46	0.21 - 0.98	0.03	0.44	0.19 - 1.05	0.03	0.20-	9	28	0.83	0.22 - 3.10	0.79	0.63	0.12 - 3.25	0.5

Total carotenes [TCR] (*μ*mol/L)	<0.31	49	93	1.00	Reference		1.00	Reference		<0.65	11	28	1.00	Reference		1.00	Reference	
	0.31-0.54	53	94	0.79	0.44 - 1.42		0.71	0.38 - 1.34		0.65-1.20	18	28	1.60	0.51 - 5.07		1.56	0.43 - 5.67	
	0.55-0.94	37	94	0.62	0.32 - 1.19		0.53	0.26 - 1.08		1.21-1.90	10	28	0.81	0.22 - 3.02		0.64	0.14 - 2.99	
	0.95-	24	94	0.35	0.17 - 0.74	0.01	0.29	0.13 - 0.67	0.00	1.91-	9	28	0.69	0.18 - 2.74	0.27	0.66	0.13 - 3.41	0.32

*β*-Cryptoxanthin [CR] (*μ*mol/L)	<0.08	49	93	1.00	Reference		1.00	Reference		<0.19	11	28	1.00	Reference		1.00	Reference	
	0.08-0.14	48	94	0.64	0.34 - 1.23		0.56	0.28 - 1.13		0.19-0.30	13	27	1.07	0.31 - 3.64		1.25	0.32 - 4.86	
	0.15-0.30	41	93	0.59	0.29 - 1.20		0.57	0.27 - 1.22		0.31-0.48	11	29	0.77	0.22 - 2.73		0.83	0.21 - 3.34	
	0.31-	25	95	0.39	0.18 - 0.85	0.03	0.32	0.13 - 0.78	0.03	0.49-	13	28	1.16	0.31 - 4.39	0.96	1.00	0.22 - 4.48	0.74

Zeaxanthin&lutein [ZL] (*μ*mol/L)	<0.64	55	93	1.00	Reference		1.00	Reference		<0.70	13	27	1.00	Reference		1.00	Reference	
	0.64-0.86	35	94	0.53	0.29 - 0.95		0.43	0.22 - 0.81		0.70-1.00	18	29	0.92	0.33 - 2.58		1.40	0.39 - 5.00	
	0.87-1.14	29	93	0.34	0.17 - 0.68		0.34	0.17 - 0.70		1.01-1.41	10	28	0.53	0.16 - 1.73		0.30	0.07 - 1.32	
	1.15-	44	95	0.70	0.37 - 1.33	0.21	0.66	0.33 - 1.35	0.24	1.42-	7	28	0.28	0.06 - 1.21	0.06	0.29	0.05 - 1.60	0.03

Canthaxanthin [CX] (*μ*mol/L)	<0.020	37	82	1.00	Reference		1.00	Reference		<0.029	15	27	1.00	Reference		1.00	Reference	
	0.020-0.030	50	104	0.96	0.53 - 1.76		1.19	0.62 - 2.28		0.029-0.036	10	26	0.56	0.19 - 1.71		0.59	0.16 - 2.10	
	0.031-0.043	37	90	0.85	0.43 - 1.69		0.91	0.43 - 1.93		0.037-0.051	11	25	0.61	0.20 - 1.90		0.52	0.14 - 1.87	
	0.044-	39	99	0.51	0.24 - 1.10	0.09	0.55	0.23 - 1.31	0.14	0.052-	12	34	0.37	0.11 - 1.25	0.14	0.39	0.09 - 1.62	0.21

Total xanthophylls [TXP] (*μ*mol/L)	<0.80	51	93	1.00	Reference		1.00	Reference		<1.08	14	28	1.00	Reference		1.00	Reference	
	0.80-1.12	39	94	0.63	0.35 - 1.15		0.57	0.30 - 1.09		1.08-1.51	15	28	0.80	0.26 - 2.48		0.84	0.23 - 3.09	
	1.13-1.52	39	94	0.70	0.38 - 1.30		0.65	0.34 - 1.26		1.52-2.11	12	28	0.67	0.21 - 2.14		0.56	0.15 - 2.05	
	1.53-	34	94	0.63	0.33 - 1.23	0.25	0.59	0.28 - 1.25	0.26	2.12-	7	28	0.29	0.06 - 1.27	0.12	0.30	0.06 - 1.42	0.1

Total carotenoids [TCA] (*μ*mol/L)	<1.22	51	93	1.00	Reference		1.00	Reference		<1.87	14	28	1.00	Reference		1.00	Reference	
	1.22-1.68	42	94	0.65	0.36 - 1.19		0.54	0.28 - 1.03		1.87-2.75	15	28	0.79	0.28 - 2.25		0.57	0.17 - 1.95	
	1.69-2.52	44	94	0.82	0.45 - 1.53		0.67	0.34 - 1.33		2.76-3.92	10	28	0.52	0.15 - .86		0.30	0.07 - 1.26	
	2.53-	26	94	0.50	0.24 - 1.02	0.13	0.42	0.19 - 0.95	0.09	3.93-	9	28	0.37	0.09 - 1.53	0.14	0.27	0.06 - 1.34	0.32

Provitamin A [PVA] (*μ*mol/L)	<0.26	43	93	1.00	Reference		1.00	Reference		<0.71	13	28	1.00	Reference		1.00	Reference	
	0.26-0.55	59	94	1.05	0.58 - 1.89		0.98	0.52 - 1.87		0.71-1.28	18	28	1.23	0.37 - 4.10		1.17	0.24 - 5.64	
	0.56-0.96	35	94	0.76	0.39 - 1.49		0.62	0.30 - 1.31		1.29-1.87	3	28	0.14	0.02 - 0.82		0.03	0.00 - 0.34	
	0.97-	26	94	0.56	0.26 - 1.20	0.09	0.43	0.19 - 1.00	0.03	1.88-	14	28	0.89	0.25 - 3.21	0.51	0.79	0.15 - 4.24	0.47

Retinol [RE] (*μ*mol/L)	<2.19	50	93	1.00	Reference		1.00	Reference		<1.92	13	28	1.00	Reference		1.00	Reference	
	2.19-2.60	36	94	0.74	0.40 - 1.35		0.76	0.40 - 1.46		1.92-2.31	11	27	0.93	0.35 - 2.48		1.00	0.30 - 3.33	
	2.61-3.22	55	94	1.21	0.70 - 2.10		1.27	0.70 - 2.28		2.32-2.77	8	29	0.69	0.22 - 2.19		1.10	0.30 - 4.01	
	>3.23	22	94	0.46	0.22 - 0.95	0.23	0.49	0.22 - 1.08	0.42	2.78-	16	28	1.26	0.46 - 3.43	0.66	2.25	0.68 - 7.47	0.16

*β*- & *γ*-Tocopherols [BT] (*μ*mol/L)	<2.26	48	86	1.00	Reference		1.00	Reference		<2.79	17	28	1.00	Reference		1.00	Reference	
	2.26-3.00	42	100	0.67	0.38 - 1.20		0.69	0.37 - 1.30		2.79-3.61	10	22	0.66	0.24 - 1.87		0.94	0.28 - 3.14	
	3.01-4.06	40	95	0.73	0.40 - 1.32		0.74	0.40 - 1.39		3.62-4.96	13	33	0.47	0.17 - 1.31		0.62	0.20 - 1.85	
	4.07-	33	94	0.56	0.29 - 1.08	0.12	0.66	0.33 - 1.31	0.28	4.97-	8	29	0.27	0.08 - 0.93	0.04	0.46	0.13 - 1.70	0.24

*α*-Tocopherol [AT] (*μ*mol/L)	<17.54	42	90	1.00	Reference		1.00	Reference		<21.39	13	27	1.00	Reference		1.00	Reference	
	17.54-21.44	46	95	0.95	0.52 - 1.73		1.00	0.52 - 1.90		21.39-26.64	20	29	1.11	0.44 - 2.84		1.40	0.45 - 4.35	
	21.45-27.46	41	95	0.79	0.41 - 1.51		0.83	0.39 - 1.77		26.65-30.75	6	28	0.25	0.06 - 1.03		0.19	0.04 - 1.05	
	27.47-	34	95	0.64	0.33 - 1.25	0.16	0.77	0.35 - 1.68	0.47	30.76-	9	28	0.40	0.11 - 1.38	0.06	0.58	0.12 - 2.78	0.25

Folic acid (ng/mL)	<3.9	23	43	1.00	Reference		1.00	Reference		<4.7	8	15	1.00	Reference		1.00	Reference	
	3.9-5.2	19	43	0.95	0.33 - 2.72		0.84	0.25 - 2.79		4.7-6.3	8	16	1.04	0.27 - 4.00		1.00	0.20 - 4.97	
	5.3-7.4	23	45	1.15	0.41 - 3.20		1.17	0.38 - 3.64		6.4-9.8	7	16	1.09	0.26 - 4.52		0.87	0.15 - 5.09	
	7.5-	16	46	0.92	0.26 - 3.28	0.99	0.82	0.20 - 3.35	0.95	9.9-	6	16	0.79	0.13 - 4.71	0.84	0.93	0.10 - 8.50	0.88

Total cholesterol [TC] (mmol/L)	<4.22	39	90	1.00	Reference		1.00	Reference		<4.66	16	27	1.00	Reference		1.00	Reference	
	4.22-4.80	38	92	0.78	0.44 - 1.38		0.76	0.42 - 1.37		4.66-5.40	17	26	1.03	0.39 - 2.70		0.92	0.33 - 2.60	
	4.81-5.45	53	95	1.27	0.74 - 2.19		1.19	0.67 - 2.11		5.41-6.00	8	28	0.45	0.15 - 1.39		0.42	0.13 - 1.42	
	5.46-	29	93	0.71	0.37 - 1.34	0.71	0.72	0.37 - 1.40	0.71	6.01-	7	27	0.35	0.11 - 1.18	0.04	0.34	0.10 - 1.17	0.04

Controls were not precisely divided into 3 even groups due to identical measurement values.

* : Adjusted for age, participating institution and smoking using conditional logistic models.

†: Adjusted for age, participating institution, smoking, alcohol drinking, body mass index and serum total cholesterol levels using conditional logistic models.

For men, risk further adjusted for smoking and other covariates (model 2:OR2) was significantly or marginally significantly lower for the highest serum levels of AC, BC, LY, TCR, CR, TCA, PVA and RE than for the lowest levels, and the OR was significantly or marginally significantly lower for moderate serum levels of ZL, TCR, TXP and TCA than for the lowest levels.

Analysis of data for women produced findings similar to those obtained for men except for BC, CR, and RE. For women, risk adjusted for smoking and other potential confounders also tended to be lower for the highest quartile of AC, LY, TCR, ZL, CX, TXP, TCA, BT, AT, and TC than for the lowest quartile, but the differences were not even marginally significant. Also for women, the OR were significantly or marginally significantly lower for moderate serum levels of BC, PVA and AT than for the lowest quartile. In both models, the OR was not lower for moderate serum RE levels than for the lowest quartile.

## DISCUSSION

In this study, all deaths that occurred during the follow-up conducted from 1988 to 1999 were included, and the study was conducted using a nested case-control design by sex, controlling for age, participating institution and smoking status. Differences between sexes and participating institutions among the present controls for serum levels of carotenoids, RE and tocopherols were approximately similar to those seen in other populations.^20^^-^^22^ Serum levels of carotenoids such as AC, BC, CR, and ZL were lower among current smokers and regular alcohol drinkers in the present study (data not shown), a finding also reported in other investigations.^9^^,^^10^^,^^20^ In addition, serum levels of carotenoids and tocopherols are associated with BMI, and serum TC levels closely correlate with serum levels of carotenoids, because serum carotenoids are carried by lipoprotein in the blood.^23^ The present study is a nested case-control study adjusted for participating institution. We tested for differences in serum levels of carotenoids and other components between cases and controls, and estimated the risk of lung cancer death associated with these serum levels, controlling for age, participating institution, smoking, alcohol consumption, BMI and serum cholesterol level, using ANCOVA and logistic regression analysis.

In the present study, analyses indicated that for men, higher serum levels of carotenoids such as AC, BC, LY, TCR, CR, ZL, TXP, and TCA were significantly or marginally significantly associated with lower lung cancer mortality, as same as previous study,^8^ whereas no such clear relationships were observed for women. Previous studies have demonstrated that serum levels of BC are lower in lung cancer cases.^8^^,^^24^^,^^25^ In the present study, there was no decreased the OR for women with the highest serum BC levels (1.21+ *μ*mol/L), although the OR tended to be lower for both men and women with moderate serum BC levels. Although the mechanisms of carcinogenesis are complex, BC (which has particularly high provitamin A activity) is considered to be a crucial factor.

The finding that BC has antioxidant activity and enhances immunity related to carcinogenesis also suggests that BC protects against oxidative stress such as damage to cell membranes, enzymes and nucleic acids caused by activated oxygen species and free radicals.^23^^,^^26^ According to some reports, most carotenoids possess antioxidant activity^23^^,^^27^^,^^28^ and carotenoids such as AC and BC can enhance cell-mediated immune responses.^29^ That is consistent with the inverse association between lung cancer death and high serum levels of AC and BC observed among men in the present study. There have been reports indicating that higher serum levels of carotenoids other than BC obtained through high intake of vegetables and fruits are associated with lower risk of lung cancer.^7^^,^^30^ High intake of vegetables and fruits can increase serum levels of carotenoids by about a few tenths of a percent, and biofactors such as vitamins and carotenoids (including AC and BC) appear to play a role in prevention of cancer incidence.^25^ In a study of bioactivity of AC, the promotion stage of lung carcinogenesis in mice was found to be suppressed more effectively by AC than BC.^31^

In contrast, intervention trials have found that high-dose administration of synthetic BC is associated with an increased incidence of lung cancer in smokers^11^ and industrial workers.^12^ A trial conducted by American physicians found no inverse association between synthetic BC administration and lung cancer incidence.^13^ A high dose of synthetic BC elevates serum BC levels more than 10-fold, and then produces prooxidant activities in biological systems.^4^^,^^32^ It has been reported that synthetic BC administration also increases levels of cell proliferation indicators such as *c-jun* and *c-fos* proteins in the lungs of ferrets.^33^ Thus, the available data suggest that high-dose administration of synthetic BC alone is associated with high risk of lung cancer incidence.^4^^,^^34^

In most Japanese populations, serum BC levels are more than 2-fold higher for women than for men.^20^^-^^22^ Serum BC levels have been shown to reflect high intake of colored vegetables, and BC consumed together with fat is incorporated effectively into the body.^35^ We previously found that, after synthetic BC administration in Japanese, serum levels of thiobarbituric acid-reactive substances (TBARS; a class of lipid peroxides) were significantly elevated and were more than 3-fold greater than serum BC levels.^36^ In the present study, the non-lower OR for women with the highest serum BC levels may be due in part to prooxidant effects of BC.

In the present study, the OR was not lower for women with higher serum CR levels, but the OR was significantly lower for men with high serum CR levels. Mandarin juice, which is rich in CR, has chemo-preventive effects against mouse lung tumorigenesis.^37^ In follow-up studies, high serum levels of CR have been associated with reduced risk of lung cancer.^7^^,^^30^ In light of these reports, our finding that serum CR levels have an inverse association with lung cancer mortality should be followed up, because of the potential for application of this protective substance in prevention of lung cancer mortality.^38^ Further study, with a large study population for women and measurement of serum levels of other carotenoids, is needed to clarify the present discrepancy between sexes.

In the present study, although serum CX was at trace levels, serum CX levels tended to be inversely associated with lung cancer risk in both sexes, and high serum ZL levels were associated with lower risk. CX and ZL also possess antioxidant activities and immune-promoting function.^25^^,^^28^^,^^29^ It has been reported that the fine bioavailability of lutein, a major carotenoid in green-leaf vegetables, is 5 times higher than that of BC.^39^ In addition, in animal experiments, CX has been shown to exhibit anticarcinogenetic activities via induction of apoptosis.^40^ These findings are consistent with the results of the present study.

In contrast, there have been no reports of clear associations between lower OR for lung cancer and higher serum levels of folic acid. It has been shown that folic acid scavenges free radicals,^41^ and that high intake of folate helps reduce the risk of lung cancer.^42^^,^^43^ An association between low mortality from lung cancer and high intake of folate has been found in a Netherlands cohort study.^43^ However, the Alpha-Tocopherol Beta-Carotene Cancer Prevention (ATBC) Study found no significant association between lung cancer incidence and serum levels of folate among elderly men.^44^ We found that serum folic acid levels were not clearly inversely associated with lung cancer death in men or women, as same as previous study.^8^ However, further investigation is needed to clarify this issue; the sample size for serum folic acid in the present study was limited.

No associations have been found between the risk of lung cancer and intake of vitamin E (AT) or vitamin A (RE).^42^^,^^45^ In 9 population studies, all cases of lung cancer death were found to have low serum BC levels, compared to surviving lung cancer patients, whereas only a few cases of lung cancer death had lower serum levels of RE and AT.^46^ In the present study, trends in serum RE levels differed between sexes; the OR for men decreased at serum RE levels greater than 3.23 *μ*mol/L. However, the OR for men did not decrease at serum RE levels less than 3.22 *μ*mol/L, which is similar to the trend for women with the highest serum RE levels. In a previous follow-up study of Japanese subjects, we found that higher serum RE levels were not significantly associated with mortality from cancer of all sites,^47^ a finding similar to the present results for women. The available evidence suggests that higher serum RE levels (more than 3.2 *μ*mol/L) reduce the risk for lung cancer by preventing certain processes of carcinogenesis. In the present study, serum TC levels were inversely associated with mortality from lung cancer, especially for women, after controlling for smoking and other covariates, a finding consistent with previous reports.^48^^,^^49^ As RE (vitamin A) and cholesterol have important biological activities related to growth factors and sex hormones, the suggested sex differences found in the present study should be also further analyzed to assess the effects of smoking^50^^,^^51^ and hormone-related metabolism.^51^

In conclusion, the present results indicate that serum carotenoids such as AC, BC LY, CX and CR are associated with reduced risk of death from lung cancer, especially for men. In addition, the risk for lung cancer appears to be lower for men with higher serum RE levels, but not lower for women with the highest serum BC levels. Further study with a large number of women cases needs to clarify the discrepancy between sexes. Serum levels of carotenoids such as AC and BC appear to be particularly promising as biomarkers to predict mortality of lung cancer in Japanese inhabitants.

## MEMBER LIST OF THE JACC STUDY GROUP

The present investigators involved, with the co-authorship of this paper, in the JACC Study and their affiliations are as follows: Dr. Akiko Tamakoshi (present chairman of the study group), Nagoya University Graduate School of Medicine; Dr. Mitsuru Mori, Sapporo Medical University School of Medicine; Dr. Yutaka Motohashi, Akita University School of Medicine; Dr. Ichiro Tsuji, Tohoku University Graduate School of Medicine; Dr. Yosikazu Nakamura, Jichi Medical School; Dr. Hiroyasu Iso, Institute of Community Medicine, University of Tsukuba; Dr. Haruo Mikami, Chiba Cancer Center; Dr. Yutaka Inaba, Juntendo University School of Medicine; Dr. Yoshiharu Hoshiyama, University of Human Arts and Sciences; Dr. Hiroshi Suzuki, Niigata University School of Medicine; Dr. Hiroyuki Shimizu, Gifu University School of Medicine; Dr. Hideaki Toyoshima, Nagoya University Graduate School of Medicine; Dr. Kenji Wakai, Aichi Cancer Center Research Institute; Dr. Shinkan Tokudome, Nagoya City University Graduate School of Medical Sciences; Dr. Yoshinori Ito, Fujita Health University School of Health Sciences; Dr. Shuji Hashimoto, Fujita Health University School of Medicine; Dr. Shogo Kikuchi, Aichi Medical University School of Medicine; Dr. Akio Koizumi, Graduate School of Medicine and Faculty of Medicine, Kyoto University; Dr. Takashi Kawamura, Kyoto University Center for Student Health; Dr. Yoshiyuki Watanabe, Kyoto Prefectural University of Medicine Graduate School of Medical Science; Dr. Tsuneharu Miki, Graduate School of Medical Science, Kyoto Prefectural University of Medicine; Dr. Chigusa Date, Faculty of Human Environmental Sciences, Mukogawa Women’s University ; Dr. Kiyomi Sakata, Wakayama Medical University; Dr. Takayuki Nose, Tottori University Faculty of Medicine; Dr. Norihiko Hayakawa, Research Institute for Radiation Biology and Medicine, Hiroshima University; Dr. Takesumi Yoshimura, Fukuoka Institute of Health and Environmental Sciences; Dr. Akira Shibata, Kurume University School of Medicine; Dr. Naoyuki Okamoto, Kanagawa Cancer Center; Dr. Hideo Shio, Moriyama Municipal Hospital; Dr. Yoshiyuki Ohno, Asahi Rosai Hospital; Dr. Tomoyuki Kitagawa, Cancer Institute of the Japanese Foundation for Cancer Research; Dr. Toshio Kuroki, Gifu University; and Dr. Kazuo Tajima, Aichi Cancer Center Research Institute.

## References

[1] Block G, Patterson B, Subar A. Fruit, vegetables, and cancer prevention: a review of the epidemiological evidence. Nutr Cancer 1992; 18: 1-29. 1408943 10.1080/01635589209514201

[2] Ziegler RG, Mayne ST, Swanson CA. Nutrition and lung cancer. Cancer Causes Control 1996; 7: 157-77. 8850443 10.1007/BF00115646

[3] Le Marchand L, Hankin JH, Kolonel LN, Beecher GR, Wilkens LR, Zhao LP. Intake of specific carotenoids and lung cancer risk. Cancer Epidemiol Biomark Prev 1994; 2: 183-87. 8318869

[4] Vainio H, Rautalahti M. An international evaluation of the cancer preventive potential of carotenoids. Cancer Epidemiol Biomark Prev 1998; 7: 725-8. 9718226

[5] Michaud DS, Freskanich D, Rimm EB, Colditz GA, Spiezer FE, Willett WC, . Intake of specific carotenoids and risk of lung cancer in 2 prospective US cohorts. Am J Clin Nutr 2000; 72: 990-7. 11010942 10.1093/ajcn/72.4.990

[6] Hirayama T. Diet and cancer. Nutr Cancer 1979; 1: 67-81.

[7] Yuan JM, Ross RK, Chu XD, Gao YT, Yu MC. Prediagnostic levels of serum beta-cryptoxanthin and retinol predict smoking-related lung cancer risk in Shanghai, China. Cancer Epidemiol Biomark Prev 2001; 10: 767-73. 11440962

[8] Ito Y, Wakai K, Suzuki K, Tamakoshi A, Seki N, Ando M, . Serum carotenoids and mortality from lung cancer: a case-control study nested in the Japan Collaborative Cohort (JACC) Study. Cancer Sci 2003; 94: 57-63. 12708475 10.1111/j.1349-7006.2003.tb01352.x PMC11160274

[9] Aoki K, Ito Y, Sasaki R, Ohtani M, Hamajima N, Asano A. Smoking and alcohol drinking and serum carotenoids levels. Jpn J Cancer Res 1987; 78: 1049-56. 3119536

[10] Stryker WS, Kaplan LA, Stein EA, Stampfer MJ, Sober A, Willett WC. The relation of diet, cigarette smoking, and alcohol consumption to plasma beta-carotene and alpha-tocopherol levels. Am J Epidemiol 1988; 127: 283-96. 3257350 10.1093/oxfordjournals.aje.a114804

[11] The Alpha-Tocopherol, Beta-Carotene Cancer Prevention Study Group. The effect of vitamin E and beta carotene on the incidence of lung cancer and other cancers in men smokers. N Eng J Med 1994; 330: 1029-35.

[12] Omenn GS, Goodman G E, Thornquist MD, Balmes J, Cullen MR, Glass A, . Effects of a combination of beta carotene and vitamin A on lung cancer and cardiovascular disease. N Eng J Med 1996; 334: 1150-5. 10.1056/NEJM199605023341802 8602180

[13] Hennekens CH, Buring JE, Manson JE, Stampfer M, Rosner B, Cook NR, . Lack of effect of long-term supplementation with beta carotene on the incidence of malignant neoplasms and cardiovascular disease. N Eng J Med 1996; 334: 1145-9. 10.1056/NEJM199605023341801 8602179

[14] Ohno Y, Tamakoshi A; for the JACC Study Group. Japan collaborative cohort study for evaluation of cancer risk sponsored by Monbusho (JACC Study). J Epidemiol 2001; 11: 144-50. 11512570 10.2188/jea.11.144 PMC11735075

[15] Tamakoshi A, Yoshimura T, Inaba Y, Ito Y, Watanabe Y, Fukuda K, . Profile of JACC Study. J Epidemiol 2005;15: S4-S8. 15881191 10.2188/jea.15.S4 PMC8565873

[16] Ito Y, Nakachi K, Imai K, Hashinoto S, Watanabe Y, Inaba Y, . Stability of frozen serum levels of insulin-like growth factor-I, insulin-like growth factor-II, insulin-like growth factor binding protein-3, transforming growth factor *β*, soluble Fas, and superoxide dismutase activity for JACC Study. J Epidemiol 2005;15: S67-S73. 15881197 10.2188/jea.15.S67 PMC8565868

[17] Ito Y, Ochiai J, Sasaki R, Suzuki S, Kushuhara Y, Morimitsu Y, . Serum concentrations of carotenoids, retinol, and *α*-tocopherol in healthy persons determined by high-performance liquid chromatography. Clin Chim Acta 1990; 194: 131-44. 2128764 10.1016/0009-8981(90)90128-f

[18] Ishiwata Y, Endo N, Ikeda R, Yasuda K. Fundamental studies on the determination of serum vitamin B12 and folate using Chemilumianalyzer ACS-180. JJCLA 1995; 20: 29-37. (In Japanese)

[19] SAS Institute Inc. SAS/STAT user’s guide (ver. 6.4th ed.), Cary NC: SAS Institute, Inc. 1989.

[20] Ito Y, Sasaki R, Suzuki S, Yasui T, Hishida H, Otani M, . Serum carotenoid levels and its sex differences in the residents living in a sourthern area of Hokkaido. Vitamins (Japan) 1994; 68: 351-63. (In Japanese)

[21] Ito Y, Shimizu H, Yoshimura T, Hashimoto T, Hayakawa N, Shinohara R, . Relationship between serum levels of lipid peroxides and carotenoids among residents in Japan. Vitamins (Japan) 1997; 71: 427-34. (In Japanese)

[22] Ito Y, Suzuki K, Ichino N, Imai H, Sakamoto H, Hokama M, . The risk of Helicobacter pylori infection and atrophic gastritis from food and drink intake: a cross-sectional study in Hokkaido, Japan. Asian Pacific Cancer Prev 2000; 1: 147-56. 12718682

[23] Bendich A, Olson JA. Biological actions of carotenoids. FASEB J 1989; 3: 1927-32. 2656356

[24] van Poppel G, Goldbohm RA. Epidemiologic evidence for *β*-carotene and cancer prevention. Am J Clin Nutr 1995; 62: 1393S-402S. 7495237 10.1093/ajcn/62.6.1393S

[25] Comstock GW, Alberg AJ, Huang HY, Wu K, Burke E, Hoffman C, . The risk of developing lung cancer associated with antioxidants in the blood: ascorbic acid, carotenoids, alpha-tocopherol, selenium, and total peroxyl radical absorbing capacity. Cancer Epidemiol Biomark Prev 1997; 6: 907-16. 9367064

[26] Bendich A. Antioxidant vitamins and their functions in immune responses. *In*: Bendich A, Phillips M, Tengerdy RP, eds. Antioxidant Nutrients and Immune Functions. Plenum Press, New York and London,1990: 35-55. 10.1007/978-1-4613-0553-8_4 2181823

[27] Gerster H. Anticarcinogenic effect of common carotenoids. Int J Vit Nutr 1993; 63: 93-121. 8407171

[28] McCall MR, Frei B. Can antioxidant vitamins materially reduce oxidative damage in humans? Free Rad Biol Med 1999; 26: 1034-53. 10232849 10.1016/s0891-5849(98)00302-5

[29] Hughes DA. Effects of carotenoids on human immune function. Proc Nutr Soc 1999; 58: 713-8. 10604207 10.1017/s0029665199000932

[30] Eichholzer M, Stahelin HB, Gey KF, Ludin E, Bernasconi F. Prediction of male cancer mortality by plasma levels of interacting vitamins: 17-year follow-up of the prospective Basel study. Int J Cancer 1996; 66: 145-50. 8603802 10.1002/(SICI)1097-0215(19960410)66:2<145::AID-IJC1>3.0.CO;2-2

[31] Murakoshi M, Nishino H, Satomi Y, Takayasu J, Hasegawa T, Tokuda H, . Potent preventive action of *α*-carotene against carcinogenesis: spontaneous liver carcinogenesis and promoting stage of lung and skin carcinogenesis in mice are suppressed more effectively by *α*-carotene than by *β*-carotene. Cancer Res 1992; 52: 6583-7. 1423303

[32] Palozza P. Prooxidant actions of carotenoids in biologic systems. Nutr Rev 1998; 56: 257-65. 9763875 10.1111/j.1753-4887.1998.tb01762.x

[33] Wolf G. The effect of low and high doses of *β*-carotene and exposure to cigarette smoke on the lungs of ferrets. Nutr Rev 2002; 60: 88-90. 11908745 10.1301/00296640260042757

[34] Wang X-D, Russell RM. Procarcinogenic and Anticarcinogenic effects of *β*-carotene. Nutr Rev 1999; 57: 263-72. 10568335 10.1111/j.1753-4887.1999.tb01809.x

[35] Prince MR, Frisoli JK. Beta-carotene accumulation in serum and skin. Am J Clin Nutr 1993; 57: 175-81. 8424385 10.1093/ajcn/57.2.175

[36] Ito Y, Sasaki R, Okamoto K, Suzuki S, Yakyu K, Shinohara R, . Serum levels of carotenoids and serum lipid peroxides. Therapeutic Res 1995; 16: 69-73. (In Japanese)

[37] Kohno H, Tajima M, Sumida T, Azuma Y, Ogawa H, Tanaka T. Inhibitory effect of mandarin juice rich in *β*-cryptoxanthin and hesperidin on 4-(methylnitrosamino) -1-(3-pyridyl)-1-butanone-induced pulmonary tumorigenesis in mice. Cancer Lett 2001; 174: 141-50. 11689289 10.1016/s0304-3835(01)00713-3

[38] Byers T, Guerrer N. Epidemiologic evidence for vitamin C and vitamin E in cancer prevention. Am J Clin Nutr 1995; 62: 1385S-92S. 7495236 10.1093/ajcn/62.6.1385S

[39] van het Hof KH, Brouwer IA, West CE, Haddeman E, Steegers-Theunissen RPM, van Dusseldorp M, . Bioavailability of lutein from vegetables is 5 times higher than that of *β*-carotene. Am J Clin Nutr 1999; 70: 261-8. 10426704 10.1093/ajcn.70.2.261

[40] Chew BP, Park JS, Wong MW, Wong TS. A comparison of the anticancer activities of dietary beta-carotene, canthaxanthin and astaxanthin in mice in vivo. Anticancer Res 1999; 19: 1849-53. 10470126

[41] Joshi R, Adhikari S, Patro BS, Chattopadhyay S, Mukherjee T. Free radical scavenging behavior of folic acid: evidence for possible antioxidant activity. Free Rad Biol Med 2001; 30: 1390-9. 11390184 10.1016/s0891-5849(01)00543-3

[42] Stefani ED, Boffetta P, Deneo-Pellegrini H, Mendilaharsu M, Carzoglio JC, Ronco A, . Dietary antioxidants and lung cancer risk: a case-control study in Uruguay. Nutr Cancer 1999; 34: 100-10. 10453448 10.1207/S15327914NC340114

[43] Voorrips LE, Goldbohm RA, Brants HA, von Poppel GA, Sturmans F, Hermus RJ, . A prospective cohort study on antioxidant and folate intake and male lung cancer risk. Cancer Epidemiol Biomark Prev 2000; 9: 357-65. 10794479

[44] Hartman TJ, Woodson K, Stolzenberg-Solomon R, Virtamo J, Selhub J, Barrett MJ, . Association of the B-vitamins pyridoxal 5′-phosphate (B_6_), B_12_, and folate with lung cancer risk in older men. Am J Epidemiol 2001; 153: 688-94. 11282797 10.1093/aje/153.7.688

[45] Ocke MC, Bueno-de-Mesquita HB, Feskens EJM, van Staveren WA. Repeated measurements of vegetables, fruits, *β*-carotene, and vitamins C and E in relation to lung cancer: The Zutphen Study. Am J Epidemiol 1997; 145: 358-65. 9054240 10.1093/oxfordjournals.aje.a009113

[46] Comstock GW, Bush T, Helzlsouer K. Serum retinol, beta-carotene, vitamin E, and selenium as related to subsequent cancer of specific sites. Am J Epidemiol 1992; 135: 115-21. 1536130 10.1093/oxfordjournals.aje.a116264

[47] Ito Y, Suzuki K, Suzuki S, Sasaki R, Otani M, Aoki K. Serum antioxidants and subsequent mortality rates of all causes or cancer among a rural Japanese Inhabitants. Int J Vit Nutr Res 2002;72:237-50. 10.1024/0300-9831.72.4.237 12214561

[48] Kritchevsky SD, Kritchevsky D. Serum cholesterol and cancer risk an epidemiologic perspective. Ann Rev Nutr 1992; 12: 391-416. 1503812 10.1146/annurev.nu.12.070192.002135

[49] Siemianowicz K., Gminiski J, Stajszczyk M, Wojakowski W, Goss M, Machalski M, . Serum total cholesterol and triglycerides levels in patients with lung cancer. Int J Mol Med 2000; 5: 201-5. 10639602 10.3892/ijmm.5.2.201

[50] Prescott E, Osler M, Hein HO, Borch-Johnsen K, Lange P, Schnohr P, . Sex and smoking-related risk of lung cancer: The Copenhagen Cancer for Prospective Population Studies. Epidemiology 1998; 9: 79-83. 9430273

[51] Payne S. ‘Smoke like a man, die like a man?’: a review of the relationship between gender, sex and lung cancer. Social Sci Med 2001; 53: 1067-80. 10.1016/s0277-9536(00)00402-0 11556776

